# Gut Microbial Taxonomy and Its Role as a Biomarker in Aortic Diseases: A Systematic Review and Future Perspectives

**DOI:** 10.3390/jcm13226938

**Published:** 2024-11-18

**Authors:** Dina Neiroukh, Aida Hajdarpasic, Cagri Ayhan, Sherif Sultan, Osama Soliman

**Affiliations:** 1 Discipline of Cardiology, School of Medicine, University of Galway, H91 TK33 Galway, Ireland; dneiroukh@universityofgalway.ie (D.N.); cagri.ayhan@universityofgalway.ie (C.A.); 2 CORRIB-CURAM-Vascular Group, University of Galway, H91 TK33 Galway, Ireland; profsherifsultan@icloud.com; 3 Department of Medical Biology and Genetics, Sarajevo Medical School, University Sarajevo School of Science and Technology, 71000 Sarajevo, Bosnia and Herzegovina; aida.hajdarpasic@ssst.edu.ba; 4 Western Vascular Institute, Department of Vascular and Endovascular Surgery, University Hospital Galway, University of Galway, H91 TK33 Galway, Ireland; 5 Department of Vascular Surgery and Endovascular Surgery, Galway Clinic, Royal College of Surgeons in Ireland, Galway Affiliated Hospital, H91 HHT0 Galway, Ireland; 6 Euro Heart Foundation, 3071 Rotterdam, The Netherlands

**Keywords:** aortic disease, gut microbiome, microbial taxa, dysbiosis, biomarker, microbial diversity

## Abstract

**Background/Objectives:** Evidence of the association between the gut microbiome and cardiovascular diseases has accumulated. An imbalance or dysbiosis of this system has been shown to play a role in the pathogenesis of cardiovascular events, including aortic diseases. We aimed to elucidate the findings of the gut microbial taxonomy associated with aortic diseases and their subtypes. Furthermore, we sought to investigate whether gut microbiome dysbiosis can be used as a biomarker for aortic disease detection and to identify which species can be disease-specific. **Methods:** A systematic search was conducted using the Preferred Reporting Items for Systematic Reviews and Meta-Analysis (PRISMA) guidelines for original research papers on gut microbiome composition in patients with aortic disease, using patients without aortic disease as the control (i.e., healthy controls). The databases PubMed, Scopus, Cochrane, and Web of Science were used by employing the medical subject headings (MeSH) terms “aortic diseases”, “microbiome”,” microbiota”, and ”taxa” before August 2024. We extracted the study characteristics, study population, and gut microbiome in aortic disease, including microbiota taxa diversity and abundance, regardless of taxa level. The National Institutes of Health (NIH) Quality Assessment Tool was used to assess study quality. Data were synthesized narratively to address the heterogeneity of the studies. **Results:** In this review, twelve studies that have identified gut microbial species and their potential impact on aortic disease pathogenesis were included. The studies showed the phyla dominance of *Bacillota*, *Pseudomonadota*, *Actinomycetota*, *Bacteroidota*, and *Euryarchaeota* in aortic disease patients. We also included the taxa sequencing methods and those used to extract the microorganisms. Aortic diseases were categorized into Takayasu’s arteritis, giant cell arteritis, aortic aneurysm, and aortic dissection. Aortic disease patients had a higher rate of dysbiosis when compared to the healthy control groups, with significantly different microbiome composition. **Conclusions:** Patients with aortic disease exhibit a distinct difference between their gut microbiota composition and that of the healthy controls, which suggests a potential biomarker role of gut dysbiosis. Further exploration of the microbiome and its metagenome interface can help identify its role in aortic disease pathogenesis in depth, generating future therapeutic options. However, a unified methodology is required to identify potential microbial biomarkers in cardiovascular and cardiometabolic diseases.

## 1. Introduction

Aortic diseases comprise a spectrum of vascular disorders presenting as acute or chronic pathological states associated with increased aortic wall stress (such as systemic hypertension), with or without aortic media abnormalities [1]. Imaging studies are typically used to identify asymptomatic individuals in the early stages of the disease or those at risk of aortic disease progression [2,3]. Cardiac biomarkers of ischemia (e.g., cardiac troponin) and heart failure (e.g., natriuretic peptides) have shown clinical success, whereas aortic disease biomarkers are as yet insufficient [3].

Pursuing aortic and vascular biomarkers is relevant for determining the timing, indication of treatment, and patient outcomes [4]. Recent evidence has shown that the gut microbiome mediates the multifactorial processes of cardiovascular disease (CVD) development through its metabolites or by signaling molecules from the gut [5]. The gut microbiota is a dynamic microbial community that varies in composition, diversity, and abundance among individuals. An imbalance in the microbial number and homeostasis is called dysbiosis, and it is associated with the gain or loss of microbiome community members. Current evidence demonstrates that alteration in the diversity and composition of the gut microbiome and its metabolites contributes to the pathogenesis and progression of CVD [6]. The microbe–host communication occurs through the structural components of the bacteria or their metabolites, such as trimethylamine, bile acids, lipopolysaccharides, and short-chain fatty acids, which have distant organ effects [5]. However, no studies have systematically reviewed the association between gut microbiota and aortic diseases. Contemporary studies of CVD, such as heart failure, indicated intestinal overgrowth of pathogenic bacteria like *Campylobacter* and *Shigella*, as well as *Candida* species [7], and members of *Enterobacteriaceae* and *Streptococcus* spp. were abundant in atherosclerotic CVD patients [8].

In this review, we sought to synthesize the evidence surrounding the relationship between gut microbiota and aortic diseases by including original studies in which the gut microbiota profiles were compared between individuals with aortic disease and controls. We aimed to investigate whether the overall microbiota composition (diversity) and its relative abundance can be used as biomarkers of aortic disease.

## 2. Materials and Methods

### 2.1. Search Strategy

The reporting of this systematic review was guided by the standards of the Preferred Reporting Items for Systematic Review and Meta-Analysis (PRISMA) Statement. Data extraction was ongoing before protocol registration; hence, this review was not eligible for PROSPERO protocol registration. The literature search (Supplementary Section S1) was performed in Embase, Cochrane, PubMed, and Web of Science, employing the medical subject headings (MeSH) terms and relevant keywords combining “gut microbiome” and “aortic disease”. Original research articles assessing gut microbiota taxa diversity or abundance in aortic disease patients were included until 3 December 2023, which we subsequently updated to 12 August 2024.

### 2.2. Eligibility Criteria

This study was limited to accessible full-text papers in the English language. We included clinical studies (e.g., cohort, cross-sectional, case-control, case-series) using human adults (≥18 years) with confirmed aortic disease, whether aortic aneurysm (thoracic or abdominal), aortitis, or acute aortic syndrome. The publications were screened by two researchers (D.N. and A.H.) independently, who reviewed the titles, abstracts, and full text in an unblinded standardized manner. Any difference of opinion was discussed and resolved with a senior contributor before the final inclusion of the article.

### 2.3. Selection Criteria

This systematic review was designed to answer the following questions: (1) Does the gut microbiota differ between aortic disease patients and participants without aortic diseases (controls)? (2) What are the differences in microbial taxa between the participants and controls? (3) As a secondary objective, what are the methods of identifying patients with aortic disease using microbial taxa? To answer our research questions, articles that did not assess the microbial taxa of a human-based aortic disease were excluded (e.g., conference abstracts, unpublished work, or gray literature). PICO (population, intervention, comparison, outcome) criteria, found in Supplementary Section S2, were used to assess the eligibility of full-text studies.

### 2.4. Quality Assessment

The risk of bias and the quality of the included studies was assessed blindly by the independent reviewers (D.N. and A.H.), including the use of the National Institutes of Health (NIH) Quality Assessment Tool for Observational Cohort and Cross-Sectional Studies found in Supplementary Table S1 [9]. All relevant discrepancies were resolved by discussion until consensus was achieved between the two reviewers and the supervisor (O.S). The quality score rating was determined for each publication on the NIH, with ratings of 0–4 (poor quality), 5–10 (fair quality), and 11–14 (good/high quality).

### 2.5. Data Analysis

The literature search results were imported to EndNote 20 software, where two authors (D.N. and A.H.) independently screened and extracted the data after automatically removing duplicates. The data extracted included study characteristics (author, year of publication, study design, location), study population (total number of patients with aortic disease and number of controls, mean age at sample collection, gender, and body mass index), and gut microbiome in aortic disease (microbiota taxa diversity and abundance). To avoid controversy regarding the microbial taxonomy, we used the NCBI Taxonomy Database browser (https://www.ncbi.nlm.nih.gov/Taxonomy/Browser/wwwtax.cgi?mode=Tree&id=2&lvl=3&srchmode=1&keep=1&unlock) accessed on 14 August 2024 [10], regardless of the different common names reported in the original publications. Further extracted data included population demographics and subject characteristics, aortic disease type, disease state (healthy, control, or aortic disease), and method of diagnosing the aortic disease, if available. In this review, no attention was paid to the type or duration of medication or the diet to which the subjects were exposed. A narrative synthesis method was applied due to the diversity of the included studies. Furthermore, the technical and computational methods used for microbiota quantification were also obtained, which included sample type, sequencing method, metadata mapping, metagenome sequencing and the sequencing region, and taxonomic profiling.

## 3. Results

### 3.1. Characteristics of the Studies Included

**Figure 1:**
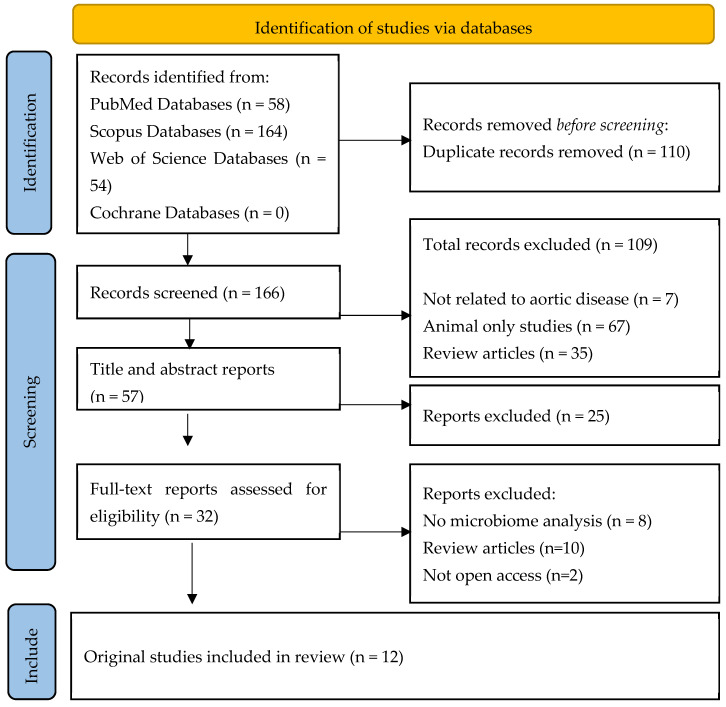
PRISMA flow diagram, which includes searches of the databases.

The literature search retrieved 276 articles, which were downloaded and uploaded to EndNote 20. After automatically extracting 110 duplicates, 166 titles and abstracts were screened. Afterwards, 57 reports were further assessed, and 32 full-text publications were screened for eligibility. Finally, 12 original studies were included in this systematic review, after excluding review articles or animal-based studies. The PRISMA chart is summarized in Figure 1 [11].

Of the twelve studies, eight were case-control studies, comprising 304 aortic disease cases and 225 healthy controls [12,13,14,15,16,17,18,19], one was a cross-sectional study, which recruited 30 participants [20], and three were genome-wide association studies (GWAS), with a total of 3950 qualified single nucleotide polymorphisms [21,22,23]. We were able to extract heterogeneous aortic diseases, ranging from aortitis and aortic aneurysm to dissection. Of the aortitis cases, three studies reported Takayasu arteritis (TAK) [12,15,17], two showed active giant cell arteritis (GCA) [17,18], and one comprised clinically isolated aortitis [17]. Aortic aneurysm (AA) was reported in five studies [14,16,20,21,22] and aortic dissections (AD) in three [13,19,23].

### 3.2. Patient Population Demographics

Most of the identified studies (Table 1) were based on the Asian population, i.e., five Chinese, two Japanese, four European, and one American study. The demographics were unavailable in the GWAS [21,22,23], the cross-sectional study [20], and one of the case-control studies [19]; therefore, we assessed the total number of aortic disease cases supplied [19]. Across the seven case-control studies that included patient demographics (age and gender), females predominated, with an overall ratio of female/male (%) of 160/144 (52.63%/47.36%) for the cases and 126/99 (56%/44%) for the controls. The average age of the aortic disease cases across the seven studies was 64.3 (±9.1) [13,14,15,16,17,18,20], and for controls, it was 61.3 (±9.7) for the six studies [13,14,15,16,17,18]. The BMI extracted in the seven studies was based on descriptive group means that were similar between the cases and controls, i.e., on average 23 (±2.2) and 24.4 (±1.7) for the cases and controls, respectively. However, the BMI of the control group was not available in one study [20].

### 3.3. Gut Microbiome Samples

The sources of gut microbiome analysis were stool, blood, and tissue. In regards to sample handling, six case-control studies collected stool samples that were packed in dry ice and stored at −80 °C before DNA extraction. The stool handled in the cross-sectional study was transferred to a medium with phosphate-buffered saline suspension. Blood samples were collected in two studies. The samples were preserved in sterile saline, along with a stabilizer solution, and in the other study, the samples were evaluated immediately after sampling. All were stored at −80 °C until analysis. Lastly, the tissue biopsy collected included an aneurysmal wall, an intraluminal thrombus, and aortic tissue.

### 3.4. Gut Microbiome Genomics and Metagenomics

The gut microbiota GWAS data was obtained from the MiBioGen results of a Finnish database; the cases were selected based on ICD-10 codes, and a Mendelian randomization design was employed. The GWAS databases included a 16S rRNA gene sequencing profile [22,23,24]. Three studies sequenced the V3–V4 regions [14,18,20], and one study used the V1–V2 region [12], of the 16S rRNA gene, using a next-generation sequencing (NGS) platform, i.e., Illumina MiSeq or Novaseq. Three studies sequenced the V3–V4 region of the bacterial 16S rDNA gene [15,17,19], and two studies used shotgun metagenomic sequencing employing Illumina Novaseq platforms [15,16]. Three studies reported the method of generating operational taxonomic units (OTUs) [12,17,18]. This is a method used for binning sequences using a divergence threshold to represent microbial individuals at different taxonomic levels, from the genus to the species level, typically with >97% similarity [25]. Meanwhile, the shotgun sequencing studies mainly used MetaPhlAn2 for taxonomic binning, which uses clade-specific major genes to differentiate microbial taxa and estimate their relative abundance (Table 2).

### 3.5. Gut Microbiota Diversity

Eight of ten studies assessed the gut microbiota diversity (alpha- and beta-diversity) between the cases and controls, whereas Nakayama et al. assessed diversity in abdominal aortic aneurysm patients only [20]. Among the alpha-diversity metrics, the number of species within a sample, the OTU, the Shannon index, and the Simpson indexes were used. The alpha-diversity metrics differed slightly or were non-significant between the cases and controls. However, one study by L. Fan et al. reported a significant Chao 1 alpha-diversity index for both the observed and rare species and T. M Getz et al. observed a significant Shannon alpha-diversity for species richness and evenness [15,18]. Meanwhile, the main beta-diversity findings (R and *p*-values) were significant (<0.05) in six out of ten studies, expressing major differences between the aortic disease and control groups [12,13]. Multiple microbial dysbiosis indexes were used to identify beta-diversity; for example, Bray–Curtis dissimilarity is based on occurrence data (abundance), while the Jaccard distance is based on presence/absence data, without abundance information. UniFrac considers the phylogenic relationships between the microbes. The unweighted UniFrac is the fraction of branch lengths between all different microbes in both samples, whereas the weighted UniFrac also includes the abundances; more details are found in Table 3.

**Figure 2:**
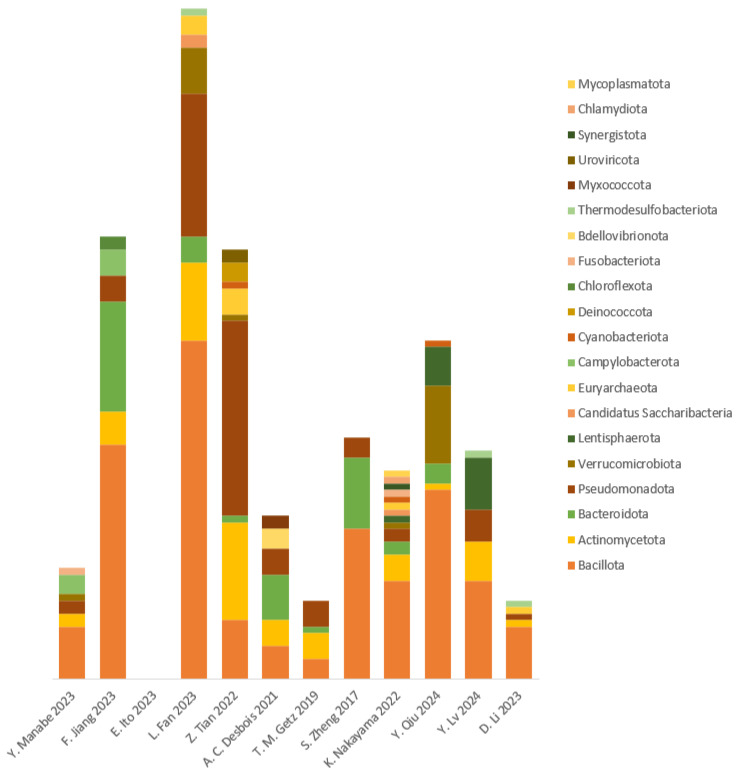
Significant and causal phyla in aortic diseases. Number of identified statistically significant (*p* < 0.05) microbiota, regardless of taxa level, used to obtain total phyla per study, arranged from most predominant (bottom) to least predominant (top) [12,13,14,15,16,17,18,19,20,21,22].

**Figure 3:**
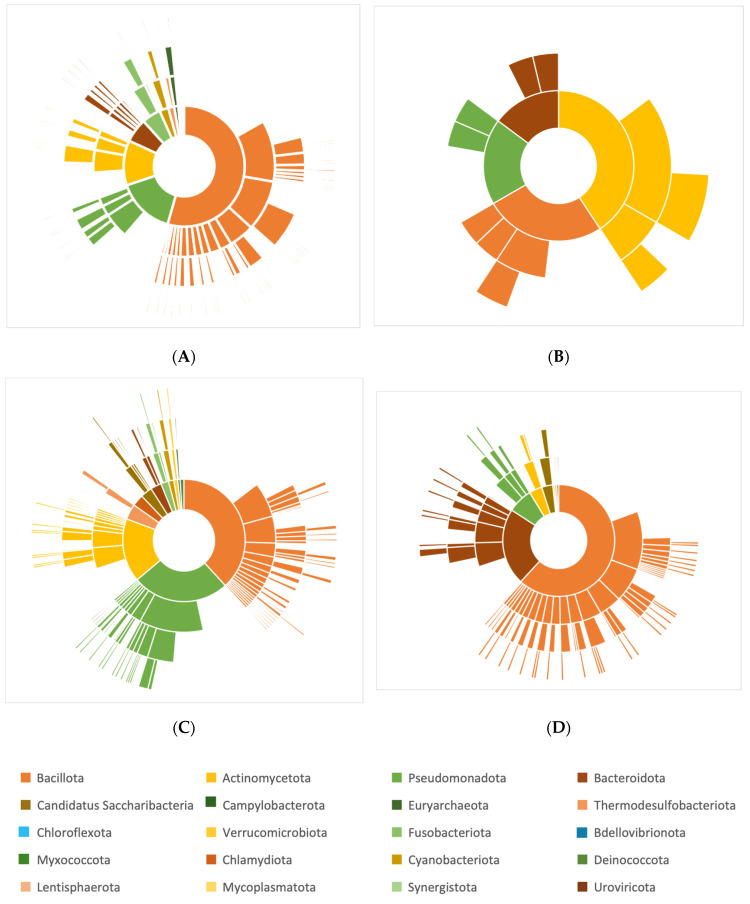
Increased abundance of the statistically significant microbial species in aortic diseases. (**A**) Microbial taxa that are TAK-specific of the *Bacillota* (39.6%) phyla were *g_Dorea* (66.7%), *g_Streptococcus* (61%), unclassified_*Lachnospiraceae* (60%), and *g_Veillonella* (100%). Of phyla *Pseudomonadota* (36.1%), g_*Haemophilus (57%);* of p_*Actinomycetota,* it was g_*Bifidobacterium* (83.3%); of p_*Verrucomicrobiota* (80%), it was g_*Akkermansia (100%)*. (**B**) The GCA was assessed in two studies for p_*Actinomycetota* (8.5%), and g_*Rhodococcus* (100%) was most GCA-specific. (**C**) Aortic aneurysms (thoracic and abdominal) were dominated by p_*Actinomycetota* (55.6%), with g_*Gordonibacter* (100%) of the *Eggerthellaceae* (100%) family and g_*Schaalia* (100%) of the *Actinomycetaceae* family (87.5%). For phyla *Pseudomonadota* (53.2%), the genus g_*Klebsiella* (76.9%) and g_*Enterobacter* (100%) of the *Enterobacteriaceae* family (74.2%) were the highest. (**D**) Aortic dissection displayed the highest *Bacillota* (42.1%) phyla among the aortic diseases, with g_*Fenollaria* (100%) being the most specific to this phylum. Of the *Bacteroidota* phyla (65.1%), g_*Bacteroides* (100%) and g_*Prevotella* (66.7%) were identified, and of p_*Pseudomonadota* (11.1%), g_*Sutterella* (60%) was observed.

The yielded studies were categorized into aortic disease patients exhibiting TAK, GCA, AA, and AD with significant taxonomic findings (*p* < 0.05), regardless of taxa level. Most studies investigating gut microbiota choose to compare aortic disease subjects to healthy controls, apart from T.M. Getz et al. and S. Zheng, who compared inflammatory thoracic aortic aneurysm (TAA) to non-inflammatory TAA and thoracic aortic aneurysm with dissection (TAAD) pre- and post-operatively, respectively [18,19]. After repeated examination of the extracted data, 20 taxonomic phyla have been identified (18 bacterial, 1 archaeon, and 1 viral) and were found to be increased in aortic disease subjects compared to the results for the controls. The most identified phyla increased in aortic disease belonged to the following: *Actinomycetota* were identified in 10 out of 12, *Bacillota* in 11/12, *Bacteroidota* in 8/12, *Pseudomonadota* in 10/12, and *Verrucomicrobiota* in 5/12 studies; more details are depicted in Figure 2. Furthermore, the previously mentioned aortic disease categories were sorted by the number of identified phyla, family, genus, and species to identify a disease-specific microbe (Figure 3).

## 4. Discussion

Our systematic review provides insights into the heterogeneity among microbiome study designs, including an overview of the differences in the computational pipelines. The studies included participants and controls of similar characteristics, such as age, participant number, and BMI. As the aortic diseases were of different phenotypes due to limited studies performed on aortic disease patients, we reported findings on statistically significant taxa that were found to be increased in the cases when compared to the results for the controls, aiming to find a correlation with metabolite production of the abundant microbial species and to identify the patterns among them. We reviewed the literature before August 2024 regarding microbiota composition in patients with aortic disease. The gut microbiota composition was consistently compared between patients with aortic disease and the controls among eight studies. Most studies in our review showed no significant difference in the number of observed species (alpha-diversity) between the groups. In contrast, a beta-diversity analysis revealed significant differences in microbiota composition.

### 4.1. Taxonomic Composition

The studies showed the phyla dominance of *Bacillota*, *Pseudomonadota*, *Actinomycetota*, *Bacteroidota*, and *Euryarchaeota*. This dysbiosis was also observed in other inflammatory diseases, such as multiple sclerosis [26], atherosclerosis [8,27], and diabetes mellitus [27]. Eleven of twelve studies found a significant increase in the phylum *Bacillota,* formerly known as *Firmicutes*. The ratio of Firmicutes (F) and Bacteroidetes (B) (F/B) is reflective of the balance of intestinal symbiotic microbiota. This was assessed by P. Petakh et al., 2023, in type 2 diabetes (T2D), with and without COVID-19, and they found that the ratio was higher in patients with both T2D and COVID-19 compared to those with only T2D or COVID-19 [28]. Additionally, it was positively correlated with C-reactive protein in T2D and COVID-19 patients, suggesting that the F/B ratio may be a potential biomarker for inflammation in these patients [28]. A study by T. Yang et al., 2015, found a decrease in microbial richness and a marked increases in the F/B in animal models with hypertension, confirming microbial dysbiosis in a small cohort of humans with hypertension [29]. Q. Yan et al., 2017, demonstrated the distribution of the opportunistic pathogenic taxa *Klebsiella* spp., *Streptococcus* spp., and *Parabacteroides merdae* in a hypertensive gut microbiome [24]. We found *Klebsiella* spp. and *Streptococcus* spp. in patients with aortic aneurysms and TAK. However, g_*Klebsiella* dominated in aortic aneurysms, whereas g_*Streptococcus* was more prominent in patients with TAK, along with the mucus-degrading bacteria s_*Akkermansia muciniphia*, s_*Bifidobacterium bifidum*.

### 4.2. Clinical Significance

Gut dysbiosis causes leakage in the epithelial barrier, leading to the translocation of bacteria and bacterial-derived components. This mechanism is induced by bacterial overgrowth in the intestine, increased intestinal permeability, and/or reduced host immunity [20]. The risk factors and etiology of aortic diseases include inflammation, high blood pressure, and atherosclerosis, which are being studied in regards to the gut microbiome. The microbial metabolites which have gained attention in CVDs include Trimethylamino-N-oxide (TMAO), bile acids, short-chain fatty acids (SCFAs), and endotoxins. A strong association with TMAO, a major adverse cardiovascular event, is noted due to atherosclerotic and thrombotic events. V.E. Brunt et al., 2020, demonstrated that the increased plasma levels resulting from TMAO promote vascular endothelial dysfunction in relation to TMAO-promoted oxidative stress [30]. Bile acids facilitate the absorption of triglycerides, cholesterol, and lipid-soluble vitamins in the intestine. The bile acid level is influenced by the gut microbiota, thereby increasing levels of circulating LDL cholesterol [31]. SCFA was observed to play an opposing role as a pro- or anti-inflammatory mediator, correlating positively with C-reactive protein, white blood cells, monocytes, and neutrophils, with a negative correlation with lymphocytes [13]. A pilot study by P. D’Aquila et al., 2021, reported that gut microbiota composition is related to various lipoprotein particles, and gut dysbiosis is associated with altered lipid metabolism and an increased expression of key genes involved in free fatty acid synthesis [32]. They also observed a positive association between bacterial blood DNA levels and serum-free fatty acids, total leukocytes, and an increased number of leukocytes and neutrophils [32]. Trained immunity can be primed by various stimuli for enhanced proinflammatory cytokine and vascular inflammation, and specific dietary strategies can intervene to attenuate aortic disease progression through the control of the circulating levels of metabolites.

### 4.3. Clinical Microbiome Studies

To the best of our knowledge, this is the first systematic review assessing the gut microbiota in aortic disease patients, evaluating the reproducibility and specificity of potential gut microbial biomarkers. The metrics and methods (i.e., sample collection) used varied across the studies, making comparison challenging, as no single index perfectly summarizes local diversity. However, we did identify that the taxa differed in their relative abundance between the aortic disease cases and the controls across two or more studies, although their role in aortic disease is largely unknown. Our findings indicate that certain aortic diseases share similar patterns of microbial changes, and that certain microbial taxa can reflect a specific aortic disease population. These findings warrant further verifications. *Pseudomonadota* was the dominant phylum identified in the studies that used shotgun metagenomic sequencing, a superior technique, as it captures most microbial genomes present within a sample at a more species-specific level [33]. Targeted 16S sequencing exhibits hypervariable regions that can differ within a single cell, impacting the identification of a unique sequence. However, it led to the adoption of database-independent operational taxonomic unit (OTU)-based methods to reduce the taxonomic resolution and allow each taxonomic unit to be treated as a distinct category, with or without taxonomic information assigned via machine learning [34,35]. The technical methods for quantifying and analyzing the gut microbiota differed. In addition, there were variations in the computational methods, including the bioinformatics pipeline used to generate OTUs and the statistical tests employed in each study. However, the studies using the NGS platform generated OTUs by clustering the 16S rRNA using 97% and 99% similarity thresholds.

## 5. Conclusions and Future Perspectives

The biomarkers for aortic diseases remain few, and there is a lack of reports detailing their role, the molecular mechanisms, and the metabolites produced by the gut microbiota. We have identified microbial taxa that are associated with specific aortic diseases. These findings present a significant opportunity to enhance our understanding of the role of gut microbiota, their metabolites, bacterial translocation, and microbiome modulation in disease development and recovery. This understanding can be leveraged to develop advanced diagnostic strategies and more personalized therapeutic approaches for patients with aortic diseases.

Sequencing methods which are specific to the bacterial species level can be more sensitive diagnostic tools for identifying increased diversity and dysbiosis. The microbiome analyses, in combination with other omics, are the basis for personalized dietary control and microbiome-targeting approaches to modulate taxa or bacterial pathways. Modifying the gut microbiota through fecal transplantation, probiotic administration, and dietary adjustments has demonstrated safety and efficacy in addressing vascular disorders such as atherosclerosis and its related risk elements. The next logical step is to conduct a randomized control trial in patients with varying stages of aortic disease, comparing gut microbiome modulation, through diet and the use of probiotics, vs. the standard of care. The primary outcome should be the rate of progression of aortic disease. In such a study, multi-omics, metagenomics, gut microbiota diversity, metabolites, and metagenomics should be analyzed.

## Figures and Tables

**Table 1:** Summary of the included studies in regards to aortic diseases and gut microbiota.

First Author	Year	Country	Aortic Disease/Control	Study Design	Diagnostic Criteria	Number of Participants (Case/Control)	Mean Age (Years) (Case/Control)	Female, n% (Case/Control)	Body Mass Index (kg/m^2^, Case/Control)
Y. Manabe et al. [12]	2023	Japan	TAK/HC	Case-control	ACR or Japanese Circulation Society	76/56	51/48 (median)	67 (88.2%)/48 (85.7%)	22.0/21.2
F. Jiang et al. [13]	2023	China	AD/HC	Case-control	NA	20/20	60.1 ± 9.91/57.85 ± 12.09	2 (10%)/2 (10%)	24.52 ± 3.66/23.15 ± 1.98 (median)
E. Ito et al. [14]	2023	China	AAA/HC	Case-control	Aneurysm diameter	30/30	75/75	6 (13%)/4 (10%)	24/23
L. Fan et al. [15]	2023	China	TAK/HC	Case-control (discovery cohort)	1990 ACR	57/40	38 ± 15/39 ± 13	44 (77.2%)/30 (75.0%)	22.5/22.2
Z. Tian et al. [16]	2022	China	AAA/HC	Case-control	ASVS guideline	33/31	68.73 ± 7.13/67.77 ± 5.04	8/10	24.56 ± 2.51/23.41 ± 2.25
A. C. Desbois et al. [17]	2021	France	LVV (GCA or TAK)/HC	Case-control	Disease activity criteria	(13 TAK; 9 GCA)/15	(45 TAK; 74 GCA)/NA	(54.5% TAK; 85% GCA)/NA	NA
			GCA (active/inactive)			6/5	77.4/70.1 (median)	3 (50%)/3 (60%)	NA
			TAK (active/inactive)			10/10	43.8/41.4 (median)	8 (80%)/9 (90%)	NA
T. M. Getz et al. [18]	2019	USA	TAA (CIA)/non-inflammatory TAA	Case-control	TAA surgery	12/23	68.5 ± 11.0/66.6 ± 8.5	9 (75%)/20 (87.0%)	20.2 ± 2.1/27.0 ± 1.3
			TAA (GCA)/non-inflammatory TAA			14/23	73.2 ± 6.8/66.6 ± 8.5	13 (92.9%)/20 (87.0%)	29.9 ± 1.9/27.0 ± 1.3
S. Zheng et al. [19]	2017	China	TAAD (pre-operative/post-operative)	Case-control	TAAD surgery	40/10	NR	NR	NR
K. Nakayama et al. [20]	2022	Japan	AAA/HC	Cross-sectional	Open AAA repair	30/NA	66.9 ± 8.9/NA	28 (93%)/NA	24.2 ± 4.2/NA
Y. Qiu et al. [21]	2024	China (Finnish database)	AA	GWAS	ICD-10	18,340/317,899	NA	NA	NA
Y. Lv et al. [22]	2024	China (Finnish database)	AA	GWAS	ICD-8, 9, 10, and NOMESCO	18,473/34,539	NA	NA	NA
D. Li et al. [23]	2023	China (Finnish database)	AD/HC	GWAS	ICD-10 codes “I71.00”, “I71.01”, and “I71.09”	18,340/349,539	NA	NA	NA

AA: aortic aneurysm; AAA: abdominal aortic aneurysm; ACR: American College of Rheumatology criteria; AD: aortic dissection; ASVS: American Society for Vascular Surgery; CIA: clinically isolated arteritis; GCA: giant cell arteritis; GWAS: genome-wide association study; HC: healthy control; ICD: International Classification of Diseases; LVV: large vessel vasculitis; n: number; NA: not applicable; NOMESCO: Nordic Medico-Statistical Committee; TAA: thoracic aortic aneurysm; TAK: Takayasu’s arteritis; TAAD: thoracic aortic aneurysm with dissection.

**Table 2:** Technical and computational methods for microbiota quantification.

First Author	Year	Aortic Disease Type	Biological Sample Type	Sequencing Method	Metagenome Sequencing, Sequence Region	Taxonomic Profiling
Y. Manabe et al. [12]	2023	TAK	Stool	rRNA	Illumina MiSeq; V1–V2	OTU with 99% similarity using QIIME 2 (v.2021.2)
F. Jiang et al. [13]	2023	AD	Stool	16S rDNA	Illumina Novaseq; V3–V4	ASVs
E. Ito et al. [14]	2023	AAA	Stool	16S rRNA	Illumina MiSeq; V3–V4	QIIME 2 (v. 2017.10) and DADA2 (v.0.99.8)
L. Fan et al. [15]	2023	TAK	Stool	Shotgun metagenomics	Illumina Novaseq; 3′ end	MetaPhlAn (v. 2.7.7) and HUMAnN2 (v.2.8.1)
Z. Tian et al. [16]	2022	AAA	Stool	Shotgun metagenomics	Illumina Novaseq; V3–V4	MetaPhlAn2 (v.2.7.7) and Kraken2 (v2.0.8)
A. C. Desbois et al. [17]	2021	LVV (GCA or TAK)	Blood	16S rDNA	Illumina MiSeq; V3–V4	Closed-reference OTU with 97% similarity using QIIME (v1.9.0)
T. M. Getz et al. [18]	2019	TAA	Tissue (aortic biopsy)	16S rRNA	Illumina MiSeq; V3–V4	Open-reference OTU with 97% similarity using QIIME (1.9)
S. Zheng et al. [19]	2017	TAAD	Stool	16S rDNA	Illumina HiSeq X; paired-end	MetaPhlAn (v2.0)
K. Nakayama et al. [20]	2022	AAA	Stool Blood Tissue (aneurysmal wall, intraluminal thrombus)	16S rRNA	Illumina MiSeq; V3–V4	QIIME (v1.8.0)
Y. Qiu et al. [21]	2024	AA	SNP (Finnish biobank)	16S rRNA	NA	Fixed or random effect IVW
Y. Lv et al. [22]	2024	AA	SNP (Finnish biobank)	16S rRNA	NA, V1–V2, V3–V4, and V4	Random effect IVW
D. Li et al. [23]	2023	AD	SNP (Finnish biobank)	16S rRNA	NA	IVW

AA: aortic aneurysm; AAA: abdominal aortic aneurysm; AD: aortic dissection; SNP: single nucleotide polymorphism; IVW: inverse variance weighted; OTU: operational taxonomic unit; TAK: Takayasu’s arteritis; TAA: thoracic aortic aneurysm; TAAD: thoracic aortic aneurysm with dissection; LVV: large vessel vasculitis; GCA: giant cell arteritis; NA: not applicable; v.: version.

**Table 3:** Gut microbiota diversity of aortic disease patients vs. controls.

First Author	Year	Aortic Disease Type	Diversity Metric (Diversity Index, Representation)	Microbial Dysbiosis Index Analysis	Main Findings (R and *p*-Values)
Y. Manabe et al. [12]	2023	TAK	α-diversity (Shannon index) α-diversity (Faith’s PD) α-diversity (observed OTUs) β-diversity (weighted UniFrac, PCoA) β-diversity (weighted UniFrac) Microbial dysbiosis index	Welch’s *t* test * Mann–Whitney U test ** Fisher’s exact test ** PERMANOVA Welch’s *t* test * Mann–Whitney U test	ns ns ns *p* < 0.05 *p* < 0.05 *p* < 0.0001
F. Jiang et al. [13]	2023	AD	α-diversity (Shannon index) α-diversity (Chao 1 index) β-diversity (Jaccard index)	Wilcoxon rank sum test Wilcoxon rank sum test ANOSIM	*p* = 0.19 *p* = 0.4 *R*^2^ = 0.251; *p* = 0.001
E. Ito et al. [14]	2023	AAA	α-diversity (PD whole tree) α-diversity (Chao 1) α-diversity (observed OTUs) α-diversity (Shannon index) β-diversity β-diversity (weighted UniFrac, PCoA) β-diversity (unweighted UniFrac, PCoA)	Mann–Whitney U test Mann–Whitney U test Mann–Whitney U test Mann–Whitney U test NR PERMANOVA PERMANOVA	ns ns ns ns ns *p =* 0.402 *p =* 0.829
L. Fan et al. [15]	2023	TAK	α-diversity (number of species) α-diversity (Chao 1 index) β-diversity (NMDS) β-diversity (Bray–Curtis)	Wilcoxon’s rank-sum test Wilcoxon’s rank-sum test Adonis MANOVA	*p =* 0.037 *p =* 0.037 R^2^ = 0.024; *p* = 0.016 *p* < 0.01
Z. Tian et al. [16]	2022	AAA	α-diversity (richness: Shannon index) α-diversity (abundance: Simpson index) α-diversity (richness: Chao 1 index) β-diversity (Bray–Curtis, PCoA) β-diversity (Bray–Curtis)	Wilcoxon’s rank-sum test Wilcoxon’s rank-sum test Wilcoxon’s rank-sum test PERMANOVA ANOSIM	ns ns *p* = 0.042 †; *p* = 0.022 ‡; *p* = 0.018 †† *p* = 0.001 *p* = 0.001
A. C. Desbois et al. [17]	2021	TAK GCA LVV	Abundance (LEfSe) α-diversity (Faith’s PD whole tree) α-diversity (Shannon index) β-diversity (weighted UniFrac) β-diversity (unweighted UniFrac)	Wilcoxon’s rank-sum test Student’s *t*-tests Monte Carlo *t*-test Mann–Whitney U tests ANOSIM	*p* < 0.05 NA NA NA NA
T. M. Getz et al. [18]	2019	TAA/non-inflammatory	α-diversity (Shannon diversity index) β-diversity (unweighted UniFrac)	DESeq2 PCoA	*p* = 0.018 *p* = 0.024
		GCA/CIA	α-diversity (Shannon diversity index) β-diversity (unweighted UniFrac)	DESeq2 PCoA	*p* > 0.7 *p* > 0.7
		Aorta/temporal arteries	β-diversity (unweighted UniFrac)	PCoA	R^2^ = 0.06; *p* = 0.0002
		Non-inflammatory aortas/non-inflammatory temporal arteries	β-diversity (unweighted UniFrac)	PCoA	R^2^ = 0.11; *p* = 0.001
		GCA-affected aorta/GCA-affected temporal arteries	β-diversity (unweighted UniFrac)	PCoA	R^2^ = 0.07; *p* = 0.001
S. Zheng et al. [19]	2017	TAAD (pre-operative vs. post-operative)	α-diversity (Simpson’s test) β-diversity (PCA)	Student’s *t*-test Spearman’s rank test	NR (slight change) *p* < 0.05
K. Nakayama et al. [20]	2022	AAA	α-diversity (richness: Shannon index) α-diversity (richness: Chao 1) Gut dysbiosis (F/B)	Fisher’s exact test Fisher’s exact test NA (F/B ratio)	6.2 (4.5–7.6) 2545 (1143–4617) 39.7
Y. Qiu et al. [21] ^§^	2024	AA	NA	NA	NA
Y. Lv et al. [22] ^§^	2024	AA	NA	NA	NA
D. Li et al. [23] ^§^	2023	AD	NA	NA	NA

AA: aortic aneurysm; AAA: abdominal aortic aneurysm; AD: aortic dissection; ANOSIM: analysis of similarities; CIA: clinically isolated arteritis; F/B: Firmicutes/Bacteroidetes Ratio; GCA: giant cell arteritis; LEfSe: linear discriminant analysis effect size; LVV: large vessel vasculitis; MANOVA: multivariate analysis of variance; ns: not significant; NMDS: nonmetric multidimensional scaling; OTU: operational taxonomic unit; PD: phylogenetic diversity; PCA: principal component analysis; PCoA: principal component analysis; PERMANOVA: permutational multivariate analysis of variance; TAK: Takayasu’s arteritis; TAAD: thoracic aortic aneurysm with dissection; ^§^: genome-wide association studies; *: normally distributed data; **: non-normally distributed data; †: total microorganism richness; ‡: virus richness; ††: bacteria richness.

## Data Availability

The articles cited in this paper are available on PubMed®, Scopus®, Web of Science®.

## Supplementary Materials

The following supporting information can be downloaded at: https://www.mdpi.com/article/10.3390/jcm13226938/s1, Section S1: Search strategy in the PubMed, which was also used for Scopus, Web of Science, and Cochrane Library databases; Section S2: PICO criteria used to assess the eligibility of full-text studies; Table S1: Studies assessed using the NIH quality assessment tool for observational cohort and cross-sectional studies.

## References

[1] Bossone E., Eagle K.A. (2021). Epidemiology and management of aortic disease: Aortic aneurysms and acute aortic syndromes. Nat. Rev. Cardiol..

[2] Members W.C., Isselbacher E.M., Preventza O., Hamilton Black J., Augoustides J.G., Beck A.W., Bolen M.A., Braverman A.C., Bray B.E., Brown-Zimmerman M.M. (2022). 2022 ACC/AHA Guideline for the diagnosis and management of aortic disease: A report of the American Heart Association/American College of Cardiology Joint Committee on Clinical Practice Guidelines. J. Am. Coll. Cardiol..

[3] Suzuki T., Bossone E., Sawaki D., Jánosi R.A., Erbel R., Eagle K., Nagai R. (2013). Biomarkers of aortic diseases. Am. Heart J..

[4] Sangiorgi G., Biondi-Zoccai G., Pizzuto A., Martelli E. (2019). Commentary: Biochemical Markers for Diagnosis and Follow-up of Aortic Diseases: An Endless Search for the Holy Grail. J. Endovasc. Ther..

[5] Schroeder B.O., Bäckhed F. (2016). Signals from the gut microbiota to distant organs in physiology and disease. Nat. Med..

[6] Ahmad A.F., Dwivedi G., O’Gara F., Caparros-Martin J., Ward N.C. (2019). The gut microbiome and cardiovascular disease: Current knowledge and clinical potential. Am. J. Physiol.—Heart Circ. Physiol..

[7] Luedde M., Winkler T., Heinsen F.-A., Rühlemann M.C., Spehlmann M.E., Bajrovic A., Lieb W., Franke A., Ott S.J., Frey N. (2017). Heart failure is associated with depletion of core intestinal microbiota. ESC Heart Fail..

[8] Jie Z., Xia H., Zhong S.-L., Feng Q., Li S., Liang S., Zhong H., Liu Z., Gao Y., Zhao H. (2017). The gut microbiome in atherosclerotic cardiovascular disease. Nat. Commun..

[9] National Heart, Lung and Blood Institute Study Quality Assessment Tools. https://www.nhlbi.nih.gov/health-topics/study-quality-assessment-tools.

[10] Schoch C.L., Ciufo S., Domrachev M., Hotton C.L., Kannan S., Khovanskaya R., Leipe D., Mcveigh R., O’Neill K., Robbertse B. (2020). NCBI Taxonomy: A Comprehensive Update on Curation, Resources and Tool. Database.

[11] Page M.J., McKenzie J.E., Bossuyt P.M., Boutron I., Hoffmann T.C., Mulrow C.D., Shamseer L., Tetzlaff J.M., Akl E.A., Brennan S.E. (2021). The PRISMA 2020 statement: An updated guideline for reporting systematic reviews. bmj.

[12] Manabe Y., Ishibashi T., Asano R., Tonomura S., Maeda Y., Motooka D., Ueda J., Yanagawa M., Edamoto-Taira Y., Chikaishi-Kirino T. (2023). Gut dysbiosis is associated with aortic aneurysm formation and progression in Takayasu arteritis. Arthritis Res. Ther..

[13] Jiang F., Cai M., Peng Y., Li S., Liang B., Ni H., Lin Y. (2023). Changes in the gut microbiome of patients with type a aortic dissection. Front. Microbiol..

[14] Ito E., Ohki T., Toya N., Nakagawa H., Horigome A., Odamaki T., Xiao J.-Z., Koido S., Nishikawa Y., Ohkusa T. (2023). Impact of Bifidobacterium adolescentis in patients with abdominal aortic aneurysm: A cross-sectional study. Biosci. Microbiota Food Health.

[15] Fan L., Chen J., Pan L., Xin X., Geng B., Yang L., Wang Q., Ma W., Lou Y., Bian J. (2023). Alterations of Gut Microbiome, Metabolome, and Lipidome in Takayasu Arteritis. Arthritis Rheumatol..

[16] Tian Z., Zhang Y., Zheng Z., Zhang M., Zhang T., Jin J., Zhang X., Yao G., Kong D., Zhang C. (2022). Gut microbiome dysbiosis contributes to abdominal aortic aneurysm by promoting neutrophil extracellular trap formation. Cell Host Microbe.

[17] Desbois A.C., Ciocan D., Saadoun D., Perlemuter G., Cacoub P. (2021). Specific microbiome profile in Takayasu’s arteritis and giant cell arteritis. Sci. Rep..

[18] Getz T.M., Hoffman G.S., Padmanabhan R., Villa-Forte A., Roselli E.E., Blackstone E., Johnston D., Pettersson G., Soltesz E., Svensson L.G. (2019). Microbiomes of inflammatory thoracic aortic aneurysms due to giant cell arteritis and clinically isolated aortitis differ from those of non-inflammatory aneurysms. Pathog. Immun..

[19] Zheng S., Shao S., Qiao Z., Chen X., Piao C., Yu Y., Gao F., Zhang J., Du J. (2017). Clinical Parameters and Gut Microbiome Changes before and after Surgery in Thoracic Aortic Dissection in Patients with Gastrointestinal Complications. Sci. Rep..

[20] Nakayama K., Furuyama T., Matsubara Y., Morisaki K., Onohara T., Ikeda T., Yoshizumi T. (2022). Gut dysbiosis and bacterial translocation in the aneurysmal wall and blood in patients with abdominal aortic aneurysm. PLoS ONE.

[21] Qiu Y., Hou Y., Wei X., Wang M., Yin Z., Xie M., Duan A., Ma C., Si K., Wang Z. (2024). Causal association between gut microbiomes and different types of aneurysms: A Mendelian randomization study. Front. Microbiol..

[22] Lv Y., Shen D., Zhang G., Wang B., Wang H., Zhang J., Tang J. (2024). Causal Associations Between the Gut Microbiome and Aortic Aneurysm: A Mendelian Randomization Study. Cardiovasc. Innov. Appl..

[23] Li D., Li F., Jin J., Yang Y., Tong Q. (2023). Unraveling the Causal Nexus: Exploring the Relationship between Gut Microbiota and Aortic Dissection. Res. Sq..

[24] Yan Q., Gu Y., Li X., Yang W., Jia L., Chen C., Han X., Huang Y., Zhao L., Li P. (2017). Alterations of the Gut Microbiome in Hypertension. Front. Cell. Infect. Microbiol..

[25] He Y., Caporaso J.G., Jiang X.-T., Sheng H.-F., Huse S.M., Rideout J.R., Edgar R.C., Kopylova E., Walters W.A., Knight R. (2015). Stability of operational taxonomic units: An important but neglected property for analyzing microbial diversity. Microbiome.

[26] Chen J., Chia N., Kalari K.R., Yao J.Z., Novotna M., Paz Soldan M.M., Luckey D.H., Marietta E.V., Jeraldo P.R., Chen X. (2016). Multiple sclerosis patients have a distinct gut microbiota compared to healthy controls. Sci. Rep..

[27] Jin M., Qian Z., Yin J., Xu W., Zhou X. (2019). The role of intestinal microbiota in cardiovascular disease. J. Cell. Mol. Med..

[28] Petakh P., Oksenych V., Kamyshnyi A. (2023). The F/B ratio as a biomarker for inflammation in COVID-19 and T2D: Impact of metformin. Biomed. Pharmacother..

[29] Yang T., Santisteban M.M., Rodriguez V., Li E., Ahmari N., Carvajal J.M., Zadeh M., Gong M., Qi Y., Zubcevic J. (2015). Gut Dysbiosis Is Linked to Hypertension. Hypertension.

[30] Brunt V.E., Gioscia-Ryan R.A., Casso A.G., VanDongen N.S., Ziemba B.P., Sapinsley Z.J., Richey J.J., Zigler M.C., Neilson A.P., Davy K.P. (2020). Trimethylamine-N-oxide promotes age-related vascular oxidative stress and endothelial dysfunction in mice and healthy humans. Hypertension.

[31] Sayin S.I., Wahlström A., Felin J., Jäntti S., Marschall H.-U., Bamberg K., Angelin B., Hyötyläinen T., Orešič M., Bäckhed F. (2013). Gut Microbiota Regulates Bile Acid Metabolism by Reducing the Levels of Tauro-beta-muricholic Acid, a Naturally Occurring FXR Antagonist. Cell Metab..

[32] D’Aquila P., Giacconi R., Malavolta M., Piacenza F., Bürkle A., Villanueva M.M., Dollé M.E.T., Jansen E., Grune T., Gonos E.S. (2021). Microbiome in Blood Samples From the General Population Recruited in the MARK-AGE Project: A Pilot Study. Front. Microbiol..

[33] Malla M.A., Dubey A., Kumar A., Yadav S., Hashem A., Abd_Allah E.F. (2019). Exploring the Human Microbiome: The Potential Future Role of Next-Generation Sequencing in Disease Diagnosis and Treatment. Front. Immunol..

[34] Galloway-Peña J., Hanson B. (2020). Tools for Analysis of the Microbiome. Dig. Dis. Sci..

[35] Marcos-Zambrano L.J., Karaduzovic-Hadziabdic K., Loncar Turukalo T., Przymus P., Trajkovik V., Aasmets O., Berland M., Gruca A., Hasic J., Hron K. (2021). Applications of Machine Learning in Human Microbiome Studies: A Review on Feature Selection, Biomarker Identification, Disease Prediction and Treatment. Front. Microbiol..

