# Supplementary material for: Gut Microbial Taxonomy and Its Role as a Biomarker in Aortic Diseases: A Systematic Review and Future Perspectives
Source: J Clin Med. 2024 Nov 18;13(22):6938. doi: 10.3390/jcm13226938 (PMC11594723; doi:10.3390/jcm13226938)
Supplement: Supplementary file 1 [file jcm-13-06938-s001.zip › jcm-3300756-supplementary.pdf]

Supplementary Material

Gut Microbial Taxonomy and Its Role as a Biomarker in Aortic Diseases: A Systematic Review and Future Perspectives

Dina Neiroukh [1,2], Aida Hajdarpasic [3], Cagri Ayhan [1,2], Sherif Sultan [2,4,5] and Osama Soliman [1,2,6,*]

Affiliations

[1] Discipline of Cardiology, School of Medicine, University of Galway, H91 TK33 Galway, Ireland; dneiroukh@universityofgalway.ie (D.N.); cagri.ayhan@universityofgalway.ie (C.A.)
[2] CORRIB-CURAM-Vascular Group, University of Galway, H91 TK33 Galway, Ireland; profsherifsultan@icloud.com
[3] Department of Medical Biology and Genetics, Sarajevo Medical School, University Sarajevo School of Science and Technology, 71000 Sarajevo, Bosnia and Herzegovina; aida.hajdarpasic@ssst.edu.ba
[4] Western Vascular Institute, Department of Vascular and Endovascular Surgery, University Hospital Galway, University of Galway, H91 TK33 Galway, Ireland
[5] Department of Vascular Surgery and Endovascular Surgery, Galway Clinic, Royal College of Surgeons in Ireland, Galway Affiliated Hospital, H91 HHT0 Galway, Ireland
[6] Euro Heart Foundation, 3071 Rotterdam, The Netherlands
* Correspondence: osoliman@eurohf.org

Section S1: Database MeSH strategy applied to PubMed.

1. "aortic disease"
2. "aortic dissection" OR "acute aortic syndrome" OR "aortic aneurysm" OR "aortic arch syndrome" OR "aortitis" OR "Takayasu arteritis" OR "aortic intramural hematoma"
3. 1 or 2
4. microbiome
5. "microbiota" OR "gastrointestinal flora" OR "gut flora"
6. 4 or 5
7. 3 and 6

Section S2: PICO criteria

Population: patients with aortic disease
Intervention: gut microbiome dysbiosis in patients with aortic disease.
Comparison: we will compare patients with evidence of gut microbiome dysbiosis in aortic disease patients and those without aortic disease
Outcome 1: Detect/ diagnosing patients with aortic disease by determining gut microbiome dysbiosis.
Outcome 2: investigate methods of microbial taxa detection/ analysis.

Table S1: Studies assessed using the NIH quality assessment tool for observational cohort and cross-sectional studies

Study; Year	1. Was the research question or objective in this paper clearly stated?	2. Was the study population clearly specified and defined?	3. Was the participation rate of eligible persons at least 50%?	4. Were all the subjects selected or recruited from the same or similar populations?	5. Was the sample size justification, power description, or variance and effect estimates provided?	6. For the analysis in this paper, were the exposure(s) of interest measured prior to the outcome(s) being measured?	7. Was the timeframe sufficient so that one could reasonably expect to see an association between exposure and outcome if it existed?	8. For exposures that can vary in amount or level. Did the study examine different levels of the exposure?	9. Were the exposure measures (independent variables) clearly defined, valid, reliable, and implemented consistently across all study participants?	10. Was the exposure(s) assessed more than once over time?	11. Were the outcome measures (dependent variables) clearly defined, valid, reliable, and implemented? Consistently across all study participants?	12. Were the outcome assessors blinded to the exposure status of participants?	13. Was loss to follow-up after baseline 20% or less?	14. Were key potential confounding variables measured and adjusted statistically for their impact on the relationship? Between exposure(s) and outcome(s)?	**SUMMARY QUALITY**
Y. Manabe et al. [12]; 2023	YES	YES	YES	YES	YES	YES	YES	NA	YES	NO	YES	CD	YES	YES	Good
F. Jiang et al. [13]; 2023	YES	YES	YES	YES	YES	YES	YES	YES	YES	NA	YES	CD	NA	YES	Good
E. Ito et al. [14]; 2023	YES	YES	YES	YES	NR	NA	YES	YES	NO	NO	YES	NR	NA	YES	Fair
L. Fan et al. [15]; 2023	YES	YES	YES	YES	YES	YES	YES	YES	YES	YES	YES	NR	NA	YES	Good
Z. Tian et al. [16]; 2022	YES	YES	YES	YES	YES	YES	YES	YES	YES	NA	YES	NA	NA	YES	Good

A. C. Desbois et al. [17]; 2021	YES	YES	NR	YES	NR	NO	YES	YES	YES	YES	YES	NR	NA	YES	Fair
T. M. Getz et al. [18]; 2019	YES	YES	YES	YES	YES	NR	YES	YES	YES	NR	YES	YES	NR	YES	Good
S. Zheng et al. [19]; 2017	YES	NO	NR	YES	NO	YES	YES	YES	NO	NR	YES	NR	NR	YES	Fair
K. Nakayama et al. [20]; 2022	YES	YES	YES	YES	YES	NO	NR	YES	YES	YES	YES	NR	NA	YES	Fair
Y. Qiu et al. [21]; 2024	YES	YES	NA	NA	YES	NO	NA	NO	YES	YES	YES	YES	NA	YES	Fair
Y. Lv et al. [22]; 2024	YES	YES	NA	NA	NO	NO	NA	YES	YES	NA	YES	YES	NA	YES	Fair
D. Li et al. [23]; 2023	YES	YES	NA	NA	YES	NA	NA	YES	YES	NA	YES	NA	NA	YES	Fair

Quality was rated as 0 for poor (0–4 out of 14 questions), I for fair (5–10 out of 14 questions), or II for good (11–14 out of 14 questions); NA: not applicable, NR: not reported.
